# Supplementary material for: T-cell stimuli independently sum to regulate an inherited clonal division fate
Source: Nat Commun. 2016 Nov 21;7:13540. doi: 10.1038/ncomms13540 (PMC5121331; doi:10.1038/ncomms13540)
Supplement: Supplementary Information — Supplementary Figures 1-10 and Supplementary References [file ncomms13540-s1.pdf]

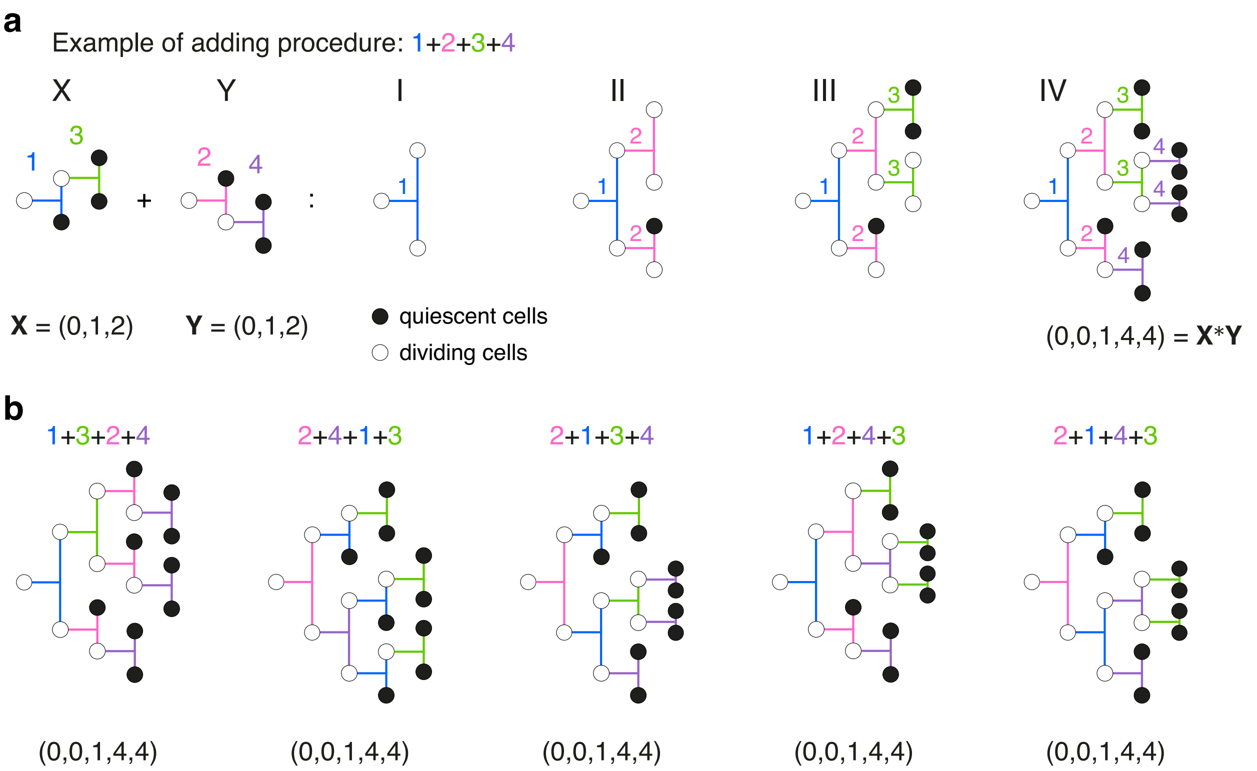

Supplementary Figure 1. Combinatorial summation of discordant family trees. The addition of discordant clonal family trees could depend on the time and place of contributing stimuli. As a result, the addition is complex as many distinct appending interlacements are needed to represent all possible stimulation orders. This is demonstrated here using an example of two clonal family trees X and Y. The open circles represent cells that will go on to divide further. The black circles represent cells that have reached DD. Thus, both families X and Y contain one progeny cell reaching DD in generation 1 and two progeny cells reaching DD in generation 2 (represented by the vectors **X** = (gen 0, gen 1, gen 2) = (0,1,2) and **Y** = (0,1,2)). (**a**) Illustrates the explicit construction of one possible addition that gives rise to one appropriate summed tree. Each clonal family tree can be broken up into multiple subsections as indicated by the different numbers and colours above (e.g. X is split into 1 and 3, blue and green respectively). These subsections of division may be programmed into the clone at the start of the response or, alternatively, a later encounter with a stimulus may be the cause of subsequent branching subsections (i.e. 3 and 4, green and purple respectively). Thus when summing discordant trees all possible permutations of subsection addition must be considered. In the worked example, the X stimulation first causes a division (I), followed by the Y stimulation (II), followed by what remains of the X stimulation (III) and finally the remainder of the Y stimulation (IV). All other possible permutations are shown in (**b**). Based upon the structure of the original clonal family trees not all orders of subsection addition are possible. Namely, the "root" of the original tree (i.e. 1 and 2) must come before the "branch" subsection (3 and 4 respectively) in the resultant summation of stimuli effects. For the remaining possible permutations the division effect of the subsection on each section of the clone is maintained irrespective of order. That is, the "root" subsection always adds onto all available arms of the clone, the "branch" subsections only add on the same side of the "root" as they were in the original tree. For this example this means that 3 can only add onto the top arm of 1 and 4 can only add to the bottom arm of 2. Based on all possible permutations of addition there are two unique branching histories generated when orientation of clonal arms are not considered. Strikingly, irrespective of the branching history the resultant division in which progeny reach DD is the same.

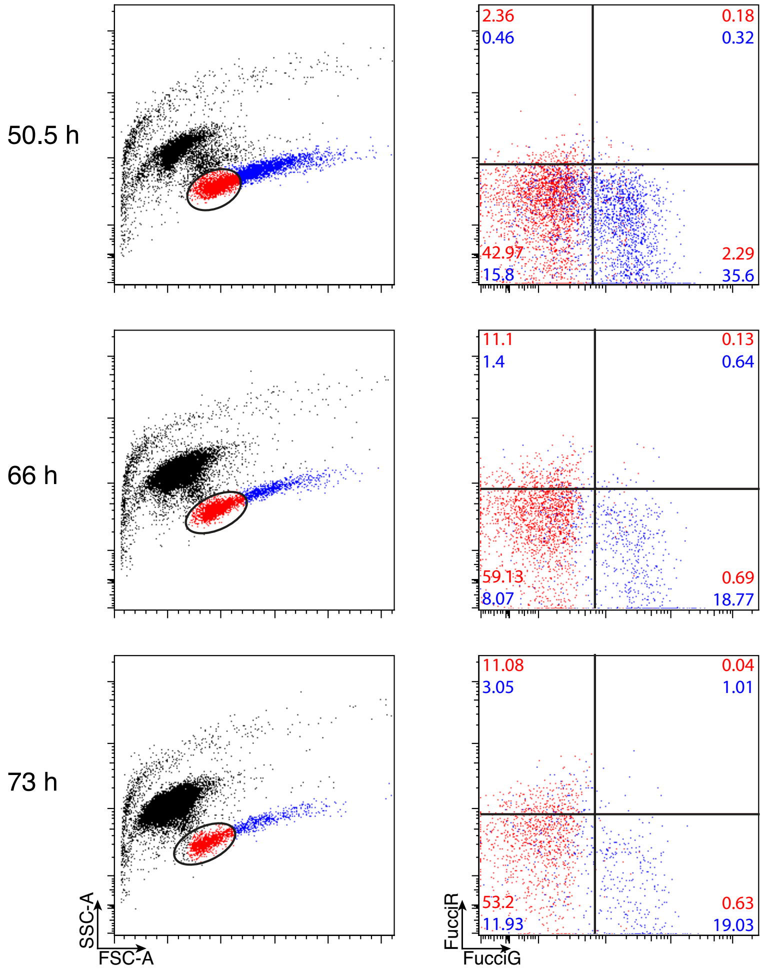

Supplementary Figure 2. Small cell size is an accurate estimator of quiescence.
CTV labelled OT-I/FucciR$^+$G$^+$ CD8$^+$ T cells stimulated with N4 peptide (0.01 µg mL^{-1}) and cultured with S4B6 (25 µg mL^{-1}) and hIL-2 (1 U mL^{-1}). FSC-A vs SSC-A profiles were used to visually determine “small” cell (red) and “large” cell (blue) gates (left column, black dots show dead cells and debris). Subsequently FucciRed *vs.* FucciGreen fluorescence was used to assess the frequency with which gating upon cell size incorrectly classifies FucciG$^+$ as small and FucciR$^+$ cells as large (right column, numbers are the average percentage of the live cell population from technical replicates). Representative of triplicate culture wells from 2 independent experiments.

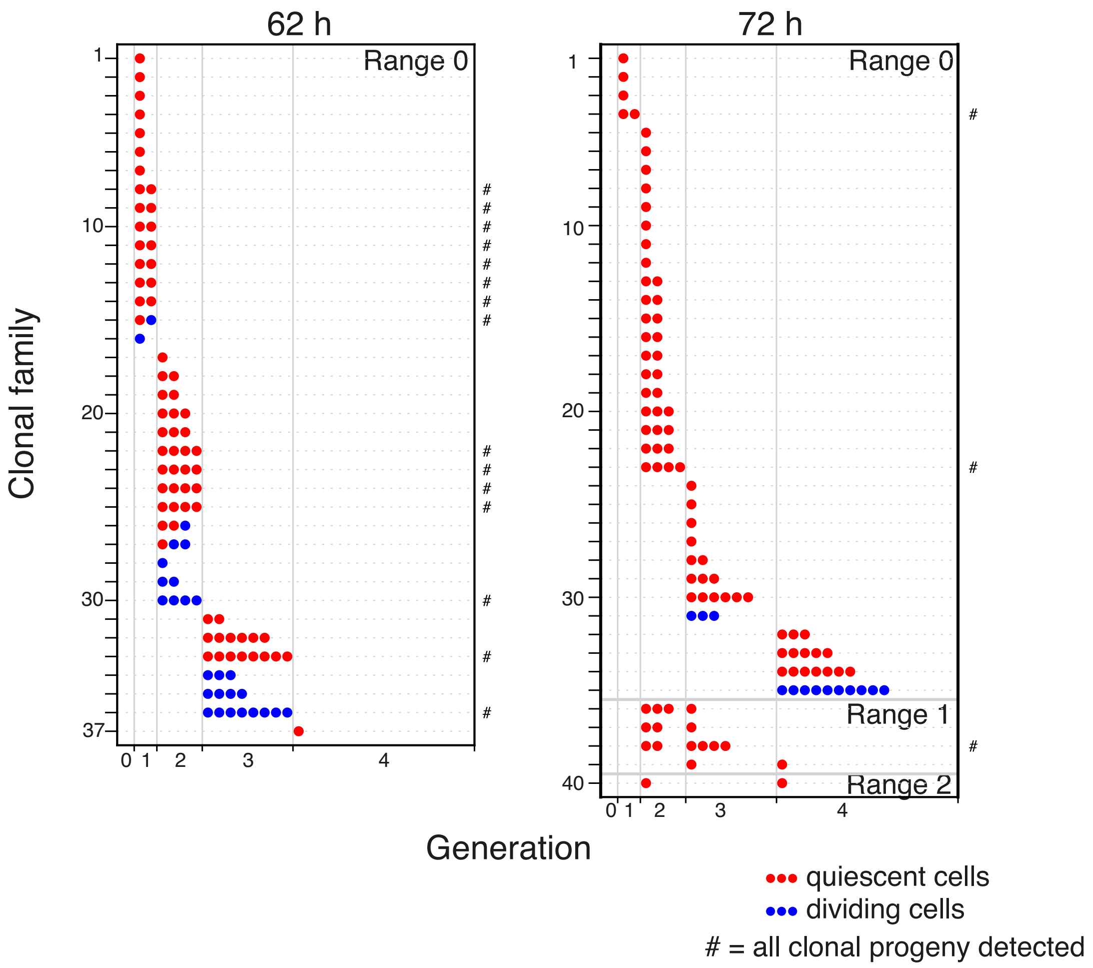

Supplementary Figure 3. Clonal T cell family proliferation is synchronized.
OT-I/*Bcl2l11*$^{-/-}$ CD8^{+} T cells were processed, stimulated and analysed as described in Fig. 2 with the exception that cells were analysed only at 62 and 72 hours. Generation number of progeny cells detected from individual clonal families at each time point from N4 + αCD28 + IL-2 stimulation condition. Progeny cells were classified as quiescent based upon small cell size (Supplementary Fig. 2 and Methods). Clonal range = maximum – minimum generation number. # denotes clones where all progeny cells were detected. Founder cell input after sorting was 168 and 168 for data from 62 and 72 hours respectively. Note that the lower clonal recovery in this experiment is likely attributable to a longer time spent out of culture during cell sorting, which reduces clone viability. All cultures contained S4B6 (25 μg mL^{-1}).

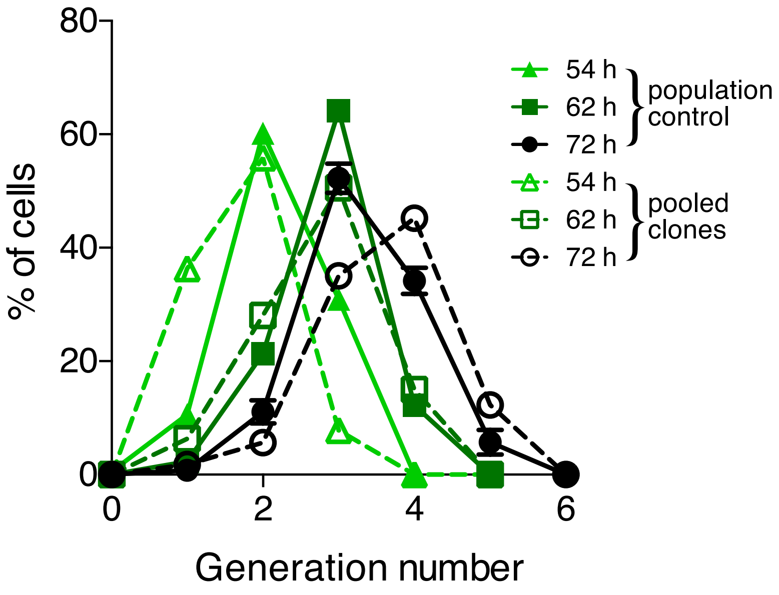

Supplementary Figure 4. Pooled clonal proliferation data recapitulates population response. OT-I/*Bcl2l11*$^{-/-}$ CD8^{+} T cells were isolated and stimulated with N4 peptide (0.01 µg mL^{-1}), αCD28 (2 µg mL^{-1}) and hIL-2 (1 U mL^{-1}) in the presence of S4B6 (25 µg mL^{-1}) as described in Fig. 2a-e and gated as outlined in Fig. 2f. At each time point the total progeny cell number detected per generation was pooled for all clones, the percentage of progeny cells per generation calculated (dotted lines) and compared to the percentage cells per generation detected in the population control (solid lines) (based on CTV dilution in 0 µM CFSE + 5 µM CTV ± 5 µM CPD labelling conditions). Graphs are representative of 2 independent experiments. Mean ± s.e.m. from triplicate culture wells.

a

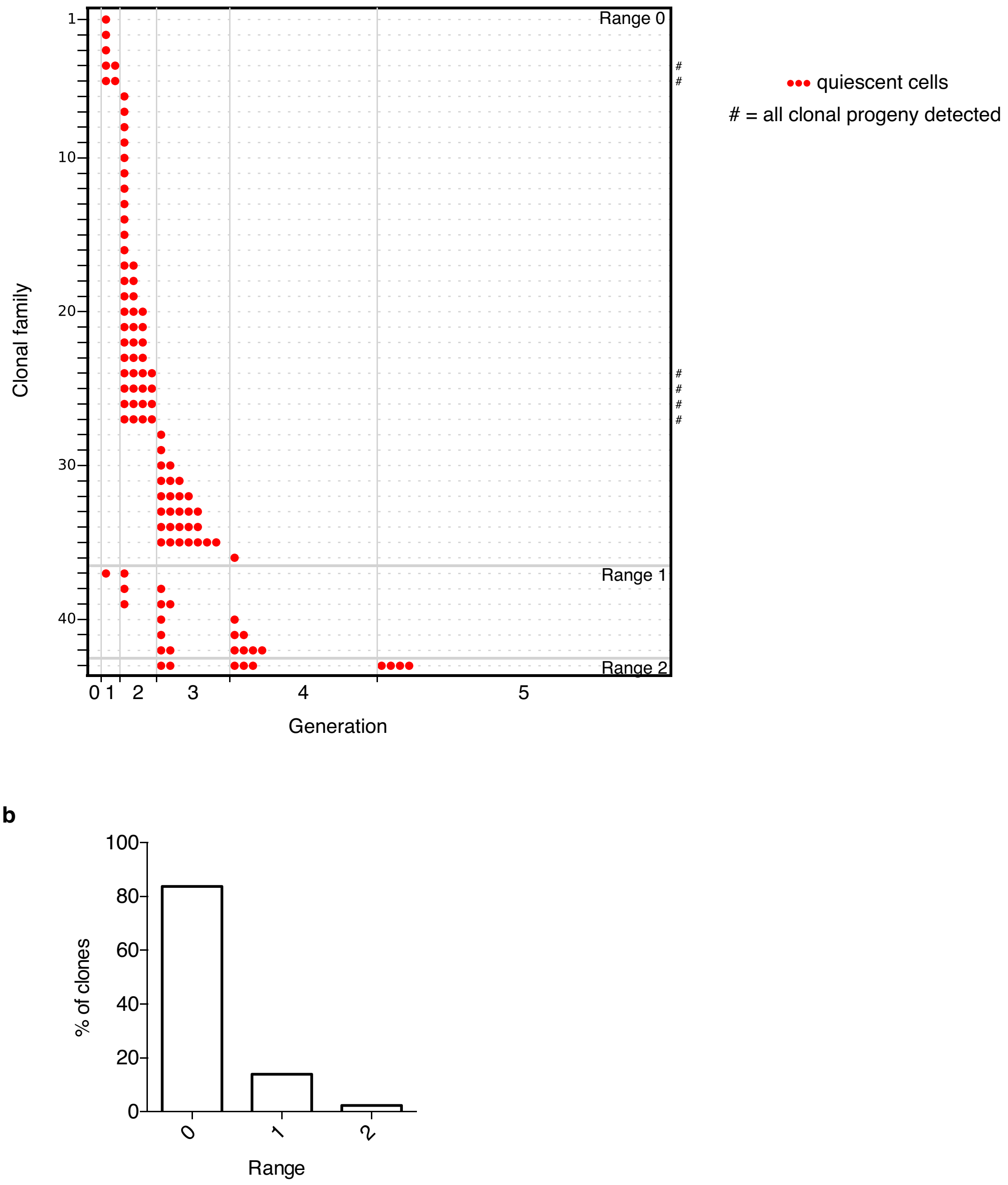

b

Supplementary Figure 5. Clonal T cell division destiny is concordant when peptide persists. OT-I/*Bcl2l11*$^{-/-}$ CD8^{+} T cells were isolated and labelled with division tracking dye combinations as outlined in Fig. 2a, b. One cell from each of the ten differentially labelled populations indicated in Fig. 2c were sorted into 96-well round-bottomed plates and 10,000 unlabelled filler cells were added to each of the sample wells. Cells were stimulated with N4 peptide (0.01 µg mL^{-1}) in the presence of S4B6 (25 µg mL^{-1}) for 50.5, 62.5 or 72.5 hours before analysis by flow cytometry as outlined in Fig. 2e. Cells were gated according to Fig. 2f only including data from the clones that were CPD^{+}, due to background autofluorescence into the CTV and CFSE channels by the unlabelled cells. (**a**) The generation in which progeny cells were detected, from clones in which all the progeny cells were quiescent. (**b**) Percentage of clones *vs.* clonal range (i.e. maxDD – minDD). Data from one experiment

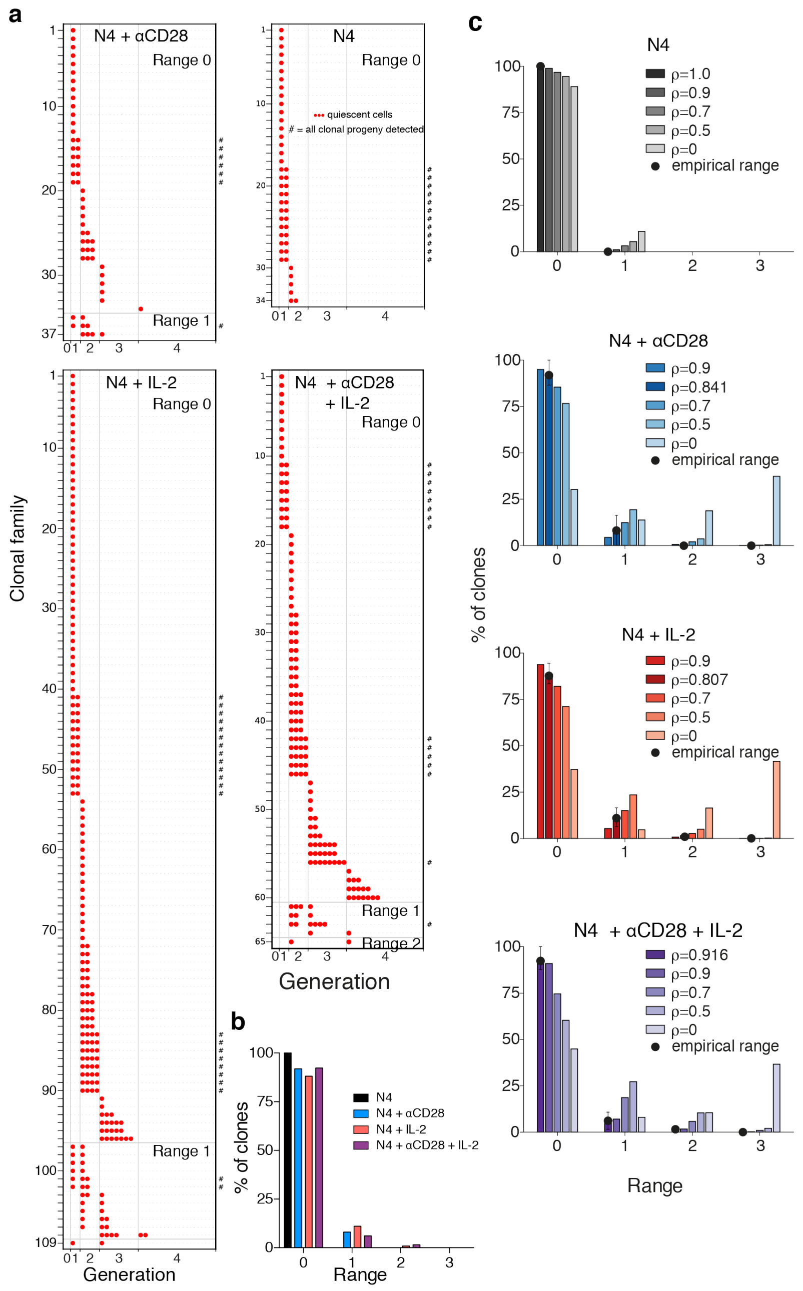

Supplementary Figure 6. Clonal family DD is highly concordant.
OT-I/*Bcl2l11*$^{-/-}$ CD8^{+} T cells labelled with a division tracking dye multiplex were stimulated with N4 peptide ± αCD28 (2 μg mL^{-1}) for 26 hours, sorted for one clone per labelling configuration per new well then cultured ± hIL-2 (1 U mL^{-1}) as described in Fig. 2. All cultures contained S4B6 (25 μg mL^{-1}). (**a**) Generation number in which progeny cells reached DD. Data pooled from 62 and 72-hours from families where all detected progeny were quiescent. Founder cell input after sorting was 168 for all conditions. (**b**) Proportion of clones with concordant (range = 0) or discordant (range > 0) DD. (**c**) To quantitatively question the level of familial correlation in DD required to explain the clonal range data in **b** a mathematical model was constructed (see Methods) parameterized by the data and pairwise correlation, ρ, in DD fate. The empirical range distribution for each condition is shown (black dots within 95% confidence intervals, see Methods), in addition to model distribution for a range of values of ρ, including the per-condition best-fit.

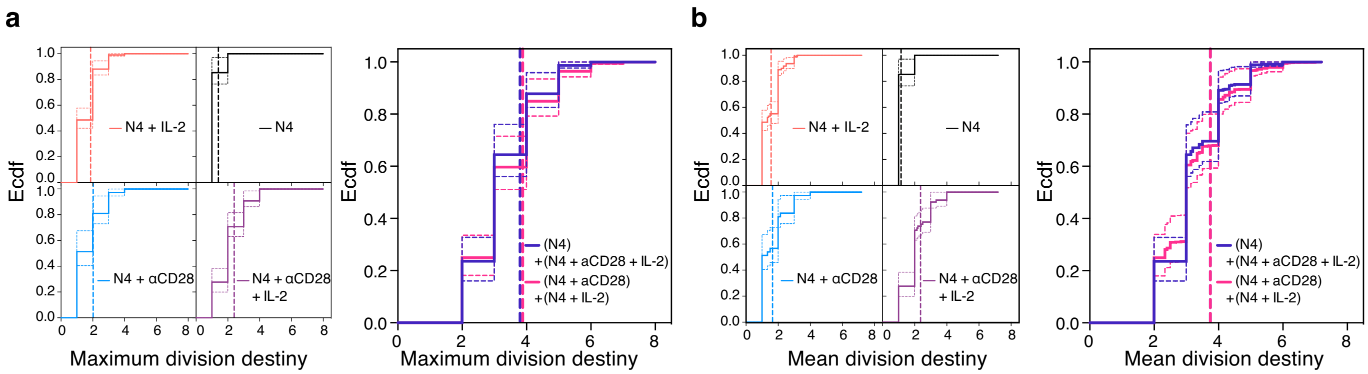

Supplementary Figure 7. Stimuli effects on DD sum independently at the level of the clonal family tree. OT-I/*Bcl2l11*$^{-/-}$ CD8^{+} T cells labelled with a division tracking dye multiplex were stimulated with N4 peptide ± αCD28 (2 μg mL^{-1}) for 26 hours, sorted for one clone per labelling configuration per new well then cultured ± hIL-2 (1 U mL^{-1}) as described in Fig. 2. All cultures contained S4B6 (25 μg mL^{-1}). Empirical cumulative distribution functions (Ecdf) of clonal (**a**) maxDD and (**b**) mDD for individual stimulation condition (left panel). To test clonal signal addition the convoluted distribution of the statistics from (N4) + (N4 + αCD28 + IL-2), with (N4 + αCD28) + (N4 + IL-2) were compared (right panel and Methods). Vertical dashed lines represent mean of the pooled clones. Dotted lines show 95% confidence intervals. A non-standard chi-square test for independence (see Methods) was not rejected for either maxDD (P=0.613) or mDD (P=0.6).

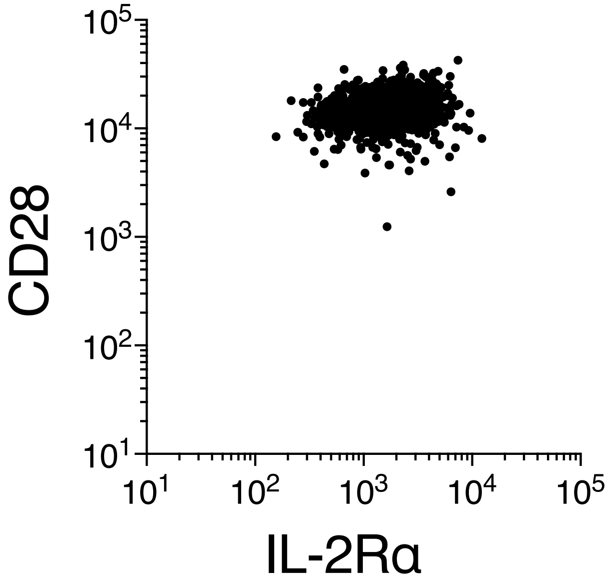

Supplementary Figure 8. CD28 and IL-2Rα levels prior to the first division are relatively uniform. CD28 and IL-2Rα expression of activated CTV labelled OT-I/*Bcl2l11*$^{-/-}$ CD8^{+} T cells stimulated for 24 hours with N4 peptide (0.01 µg mL^{-1}) in the presence of αCD28 (2 µg mL^{-1}), S4B6, (25 µg mL^{-1}) and hIL-2 (1 U mL^{-1}). Representative of duplicate culture wells from 3 independent experiments.

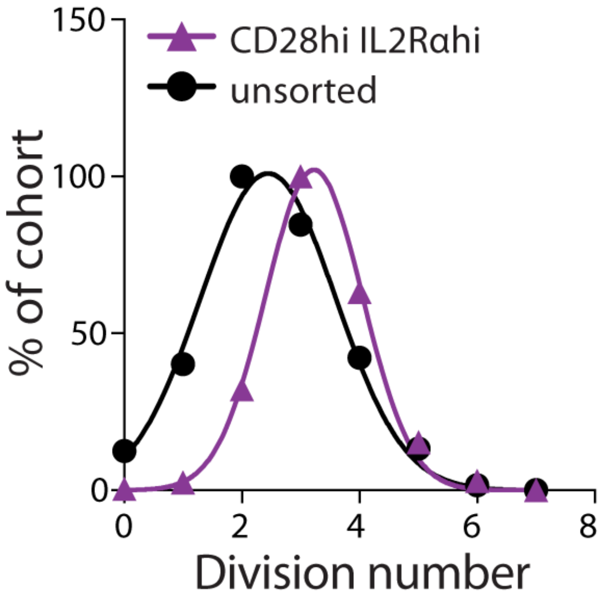

Supplementary Figure 9. Variation in DD is reduced when restricted to a constrained receptor range.

Naïve CTV labelled OT-I/*Bcl2l11*$^{-/-}$ CD8^{+} T cells were sorted for CD28^{hi} expression (top 20%) and stimulated with N4 peptide + αCD28 (2 μg mL^{-1}). After 26 hours cells were sorted for IL-2Rα^{hi} expression (top 35%) and placed back in culture with hIL-2 (3.16 U mL^{-1}). After 75 hours cell proliferation of sorted cells and unsorted control was compared. Cohort number vs division number fitted with normal distributions. Data from one experiment. Mean ± s.e.m. of triplicate culture wells.

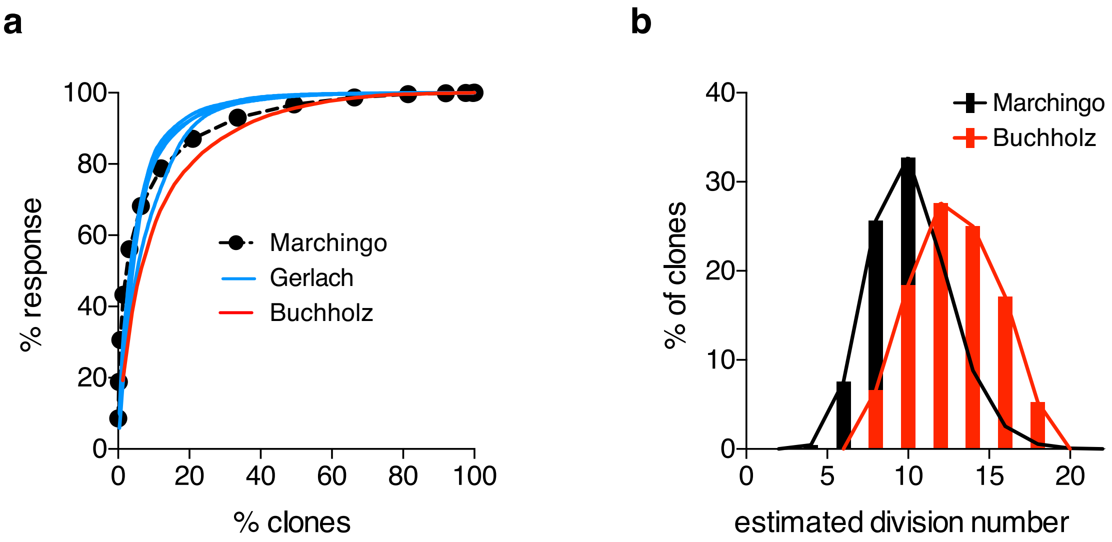

Supplementary Figure 10. Concordant T cell division destiny is consistent with previous *in vivo* population and clonal response data.
Data for the burst size of individual OT-I CD8$^+$ T cell clones at response peak during a Listeria monocytogenes-OVA infection was obtained from previous clonal studies from Gerlach and colleagues (data from Fig. 1C, clones distinguished using genetic barcoding technology [1]) and Buchholz and colleagues (data from Fig. 1E, clones distinguished using a congenic marker matrix [2]). This was compared to a previously published population time course of OT-I/FucciRG CD8$^+$ T cells responding to an HKx31-OVA influenza infection (Fig. 1D, E from Marchingo et al.[3]). The DD distribution for the OT-I/FucciRG CD8$^+$ T cells in this experiment was estimated by mathematical fitting using the Cyton model (**a**) The clonal contribution to T cell response magnitude was predicted for population response data (black circles) by assuming the clonal progeny exhibited concordant DD and followed the DD distribution estimated previously by Cyton fitting[3]. This was compared to the contribution to response magnitude of individual OT-I CD8$^+$ T cell clones from Gerlach and colleagues (blue lines, each line shows data from an individual mouse) and Buchholz and colleagues (red line). (**b**) By assuming that DD was clonally concordant the DD distribution generated by individual clonal families in Buchholz et. al. [2] was estimated from the response magnitude (see Methods) and compared to the DD distribution estimated by Cyton fitting to the population data in Marchingo et al.[3]. Note: Due to the read-count threshold that was applied to remove background noise in Gerlach et al. [1], data on small families is also lost. As a result it is not possible to estimate the full response DD distribution for this data.

Supplementary References:

1. Gerlach C., *et al.* Heterogeneous differentiation patterns of individual CD8+ T cells. *Science* **340**, 635-639 (2013).

2. Buchholz V. R., *et al.* Disparate individual fates compose robust CD8+ T cell immunity. *Science* **340**, 630-635 (2013).

3. Marchingo J. M., *et al.* Antigen affinity, costimulation, and cytokine inputs sum linearly to amplify T cell expansion. *Science* **346**, 1123-1127 (2014).
